# Age at First Gestation in Beef Heifers Affects Fetal and Postnatal Growth, Glucose Metabolism and IGF1 Concentration

**DOI:** 10.3390/ani11123393

**Published:** 2021-11-27

**Authors:** Sebastian López Valiente, Alejandro M. Rodríguez, Nathan M. Long, Graciela Quintans, Florencia E. Miccoli, Isabel M. Lacau-Mengido, Sebastian Maresca

**Affiliations:** 1 Estación Experimental Agropecuaria Cuenca del Salado, Instituto Nacional de Tecnología Agropecuaria, Rauch BA 7203, Argentina; rodriguez.alejandro@inta.gob.ar (A.M.R.); maresca.sebastian@inta.gob.ar (S.M.); 2 Department of Animal and Veterinary Sciences, Clemson University, Clemson, SC 29634, USA; nlong2@clemson.edu; 3 Instituto Nacional de Investigación Agropecuaria, Treinta y Tres 33000, Uruguay; gquintans@inia.org.uy; 4 Facultad de Ciencias Agrarias, Universidad Nacional de Lomas de Zamora, Lomas de Zamora BA 1836, Argentina; fmiccoli@agro.uba.ar; 5 Laboratorio de Regulación Hipofisaria, Instituto de Biología y Medicina Experimental (CONICET), Buenos Aires 1428, Argentina; ilacau@dna.uba.ar

**Keywords:** dam age, fetal programming, bovine, growth, offspring

## Abstract

**Simple Summary:**

In cow-calf operations, replacement heifers are bred to calve at 2 years of age or older. However, as beef production has become more intensive during the last 20 years, an increasing number of farmers have lowered the age at first service. Numerous studies have focused on determining the optimum reproductive development of beef heifers to ensure a maximum pregnancy rate. Maternal age during gestation has been suggested to be an important influence on the nutritional environment for the embryo and fetal growth. No studies have been conducted to determine the possible effects of heifer age at first gestation on fetal and postnatal growth and development. This study aimed to determine the effects of age at first gestation on offspring growth performance, glucose metabolism and insulin-like growth factor 1 concentration from birth to weaning of calves from adult cows and heifers at 15 or 27 months of service. First-breed heifers produce lighter progeny at birth than mature cows, and calves from younger heifers were lighter at weaning than calves from older heifers. Milk production was similar between heifers and lower than that from adult cows. Age at first gestation may affect offspring postnatal growth performance, glucose metabolism and IGF1 concentration.

**Abstract:**

This study aimed to determine the effects of age at first gestation on offspring growth performance, glucose metabolism, and IGF1 concentration. Heifers impregnated by AI from a single bull at 15 months of age (15 M, n = 20), or 27 months of age (27 M, *n* = 20), and multiparous cows (adult, *n* = 20) were used. Dams from all groups were managed in a single group during gestation and lactation. Gestational length was longer in the 15 M and 27 M than in adult dams (*p* = 0.009). Bodyweight at birth, at weaning and ADG during lactation were higher in calves from adult dams than in those from 27 M dams, and higher in calves from the latter than in 15 M calves (*p* < 0.001). Calves from 15 M dams had an increased head circumference/BW ratio compared to calves from 27 M dams, while calves from this latter group had an increased ratio compared to calves from adults (*p* = 0.005). Body mass index was greater in calves from adults than in those from 15 M and 27 M dams (*p* = 0.002). Milk production from 15 M and 27 M dams was similar but lower than that from adults (*p* = 0.03). Calves born from adult dams had greater blood glucose concentrations than those from 15 M and 27 M dams (*p* < 0.05). Serum IGF1 concentrations were higher in calves from adults than in calves from 15 M and 27 M dams (*p* = 0.01). This study showed that age at first gestation affects offspring postnatal growth performance, glucose metabolism and IGF1 concentration.

## 1. Introduction

Traditionally, in Argentinian cow-calf operations, replacement heifers are bred to calve at greater than 2 years of age. However, as beef production has become more intensive during the last 20 years, an increasing number of farmers have lowered the age at first service (15 to 18 months). International literature has focused on management strategies to obtain puberty at early ages [1,2]. It has been demonstrated in numerous studies that the level of nutrition influences age and bodyweight (BW) at puberty. These studies suggest that heifers need to attain, at least, 55% to 60% of their mature BW to ensure a maximum pregnancy rate [3,4]. Nevertheless, no studies have been conducted in order to determine the possible effects of heifer age at first gestation on fetal and postnatal growth and development. Maternal age during gestation has been suggested to be an important influence on the nutritional environment for the embryo and fetal growth [5,6]. The birth and weaning BW of heifer progeny are generally 10 and 12% lower, respectively, compared to progeny from mature cows [7]. Nutrient availability during fetal development may play a role in altered glucose–insulin homeostasis during early postnatal growth and development in cattle. LeMaster et al. [8] reported that late gestation nutrient restriction reduced birth weight and postnatal glucose concentrations compared to control cows. Insulin–like growth factor I (IGF1) is essential for regulation of animal growth, and nutritional restriction that affects fetal development may result in permanent alterations of the IGF axis [9,10]. Maternal nutrient restriction in mid to late gestation resulted in altered circulating levels of IGF1 in offspring [11,12]. Thus, it is highly possible that the age at first breed in beef heifers will alter fetal and postnatal growth due to differences in energy partitioning in the dam. The objective of this study was to determine the impact of maternal age at first gestation on fetal growth, lactation performance, calf growth, glucose metabolism and IGF1 concentration.

## 2. Materials and Methods

### 2.1. Animal Management

The experimental study was conducted at the Experimental Station of Cuenca del Salado INTA (Buenos Aires, Argentina). All procedures involving animal handling were approved by CICUAE INTA-CERBAS (Institutional Committee for Care and Use of Experimental Animal of South Buenos Aires Region), approval No. 161. The experiment was a complete randomized design. A total of 20 pregnant heifers at 15 months of age ± 5 days (15 M), 20 pregnant heifers at 27 months of age ± 4 days (27 M), and 20 pregnant multiparous cows (between 4 and 7 years old, adults) were selected randomly from the pregnant females at first insemination in an experimental herd for the present study. Heifers and multiparous Angus cows were fixed-time artificially inseminated (TAI) after a 7 day progesterone device (Cronipres^®^, Biogénesis-Bago, Argentina) and estrogen synchronization protocol using frozen thawed semen from a single Angus sire. At 30 days after TAI, pregnant dams were identified by transrectal ultrasound (Aquila pro, Esaote Europe B.V. Maastricht, NL; 5-MHz probe). Body condition score (BCS 1 = emaciated to 9 = obese; [13]) was assigned by a single trained technician throughout the experiment. Maternal BW and BCS were recorded on the first day of the trial (day 107), at parturition (day 0), and at weaning (day 236). Heifers and cows from the three age treatments were managed during gestation in a single group on oat pasture (IVDMD 628 g/kg; CP 159 g/kg). After calving, all cows and calves were managed in a single group on improved pastures (IVDMD 510 g/kg; CP 107 g/kg), until weaning at 236 ± 10 days of age.

### 2.2. Milk Production and Composition

Milk yield was measured by using a single-cow portable milking machine according to Quintans et al. [14] protocol, and milk production was estimated following Restle et al. [15] equation for a 24 h period. Briefly, calves were separated from their mothers at midday and were fitted with nose rings (San Miguel, Bahia Blanca, Argentina). Each cow was injected intramuscularly with a dose of oxytocin (10 IU Over^®^, Argentina) to facilitate milk letdown. After 5 min, dams were milked and placed back in the same paddock with their calves. The following morning, at approximately 0700 h, dams were milked again and milk yield was recorded with a milk meter (TrueTest, Auckland, New Zealand). A 10 mL sample of milk from every dam was taken for determination of protein, fat, lactose and total solids (IDF 141C:2000 Bentley Instruments, Chaska, MN), and urea (Chemspec 150, Bentley Instruments) analysis. Milk yield records and milk samples were taken at four different times: 32, 97, 151, and 236 days after parturition (±10 days).

### 2.3. Calf Measurement

Calf sex and BW were recorded with an electronic scale, in the first hours after calving and at weaning (day 236). The morphometric measurements recorded were: cannon bone circumference (narrowest point of metacarpus), heart girth (posterior to foreleg), height (linear distance from the floor to trochanter major of the femur), body length (linear distance along the vertebral column from the first coccygeal vertebra to the occipital bone) and head circumference (measurement collected around mandible and parietal bone just posterior to eye orbits). The ratio of newborn measurement and body weight at birth was calculated to determine if fetal growth is affected asymmetrically. To calculate the body mass index, BW at birth was divided by the square root of body length [16].

### 2.4. Blood Collection and Assays

Calves´ blood samples were taken from jugular vein at birth, 55, 98, 153, and 236 days. Samples were taken at the same time in the day after 16 h fasting and no water supply, preserved in ice for 3 h, centrifuged (2500× *g*, 15 min) for serum collection and stored at −20 °C until laboratory analysis. Glucose level was measured using an electronic glucometer (Abbott©, Berkshire, UK) immediately after the blood sample was taken [17]. Insulin concentration was assessed in serum samples by radioimmunoassay technique (RIA) using anti-bovine insulin antibody (Sigma, St. Louis, MO, USA) and standard human insulin (Laboratorios Beta, Buenos Aires, Argentina); the minimum detectable concentration was 0.05 ng/mL. Intra- and inter-assay coefficients of variation were <7.8% and 10.3%, respectively. Serum IGF1 concentration was serum quantified in one assay via RIA after acid ethanol extraction [18] using IGF1 antibody (UB2-495) of the NIDDK. Intra-assay coefficient of variation was 7.6%, and the minimum detectable concentration was 2.4 ng/mL.

### 2.5. Statistical Analyses

All the analyses were performed using dams or offspring as the experimental unit. Blood glucose, serum insulin and IGF1 levels from calves and milk variables (yield and composition) were analyzed as repeated measures using the MIXED procedure of SAS (SAS Inst., Inc., Cary, NC, USA). Treatment (15 M, 27 M, adult), calf sex and sampling time were included in the model as fixed effects and animal as a random effect. Dam BW, BCS, offspring BW and body measurements were analyzed using the MIXED procedure of SAS (SAS Inst. Inc., Cary, NC, USA). The model included the fixed effects of maternal age and calf sex and the interaction and animal as a random effect. Significant differences were considered at *p* ≤ 0.05, and tendencies declared at *p* ≤ 0.10.

## 3. Results

The effect of age at pregnancy on dam performance is shown in Table 1. Body weight was reduced in 15 M dams compared to 27 M dams, and reduced in both heifer groups compared with adult dams at the beginning of the experiment (*p* < 0.001), at calving (*p* < 0.001) and at weaning (*p* < 0.001). Body condition score (BCS) at the beginning of the experiment (*p* < 0.001) and at calving (*p* = 0.03) was increased in heifers (15 M and 27 M) compared with adult dams. At weaning, BCS was lower in 15 M dams than in adult dams, and intermediate in 27 M dams (*p* = 0.02).

Newborn body measurements, postnatal BW and ADG from birth to weaning are shown in Table 2. Gestation length was longer in 15 M and 27 M dams than in adult dams (*p* = 0.009), and no effect of sex or treatment x sex interaction were observed (*p* > 0.10). The BW at birth decreased (*p* = 0.002) as age at first calving decreased. Calves from 15 M dams weighed 2.3 kg less than calves from 27 M dams, and 5.0 kg less than those from adult cows. Evolution of BW until weaning increased as age at first calving increased (*p* < 0.001). During lactation, calves from 15 M cows gained 90 g/day less than those from 27 M cows, and the latter gained 110 g/day less than those from adult cows. Consequently, the calves from 15 M cows weighed 23 kg less than those from 27 M cows at weaning, and the latter weighed 25.3 kg less than those form adult cows at weaning (*p* < 0.001). As shown in Table 2, age of dams also affected the newborn body measurements. Head circumference, body length and height were similar between newborn calves from 15 M and 27 M dams but lower than those from adult dams (*p* < 0.05). Calves from 15 M dams had smaller heart girth than calves from 27 M and adult dams (*p* = 0.009). There was no effect from dam age on cannon bone circumference (*p* = 0.15). Heifer calves were 3.6 kg lighter at parturition (*p* = 0.001) and had reduced heart girth (2.7 cm; *p* = 0.01) and cannon circumference (0.7 cm; *p* = 0.004) compared to bull calves.

The age of dams at gestation affected fetal growth asymmetrically. Calves born from 15 M dams had higher head circumference/BW ratio than calves from 27 M dams, and this latter group had a higher ratio with respect to calves from adult dams (*p* = 0.005; Table 3). Heart girth/BW ratio (*p* = 0.01) and height/BW ratio (*p* = 0.001) were greater in calves from 15 M and 27 M dams than in calves from adult dams. Calves from 15 M dams had greater cannon bone circumference/BW ratio (*p* = 0.002) and body length/BW ratio (*p* = 0.04) than calves from 27 M and adult dams. There was no age x sex interaction for any variables (*p* > 0.10). Calves from 15 M and 27 M dams had lower body mass index than calves from adult dams (*p* = 0.002).

Calf sex influenced ratios of body measurements to birth weight. Head circumference/birth BW was increased in heifers compared to bulls (1.63 vs. 1.46, *p* = 0.0008). Heart girth/ birth BW was increased in heifer calves compared to bull calves (2.60 vs. 2.37, *p* = 0.001). Body length and height/ birth BW were both increased in heifers compared to bull calves (2.58 vs. 2.28, *p* < 0.001; and 2.54 vs. 2.26, *p* < 0.001, respectively). Body mass index was increased (*p* < 0.001) in bulls compared to heifers (3.79 vs. 3.34)

**Figure 1:**
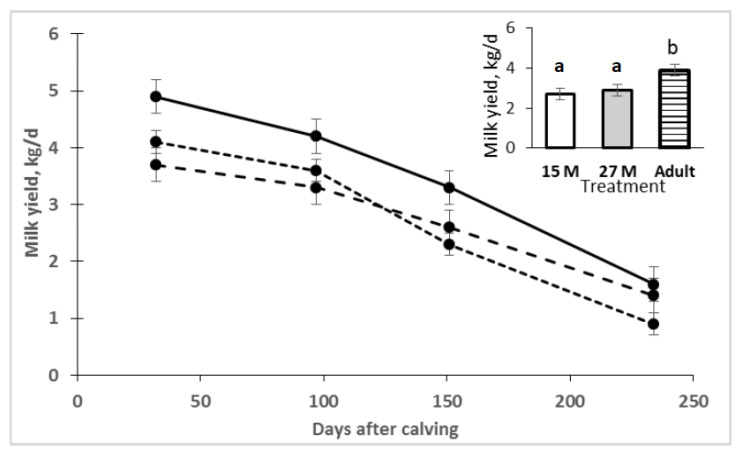
Milk production from pregnant heifers at 15 months (••••), 27 months (– – –) and multiparous cows (–––) until weaning. Different letters indicate difference between means is significant (*p* < 0.05) between treatments. Values are means ± SEM. Treatment, *p* = 0.03; time, *p* < 0.0001; treatment × time, *p* = 0.94.

Maternal milk production data are shown in Figure 1. Milk yield was lower in primiparous cows of 15 M and 27 M compared to adult cows (2.7, 2.9 and 3.9 ± 0.3 l/day, respectively; *p* = 0.03). Data for maternal milk composition are reported in Table 4. Urea and lactose concentrations were higher in primiparous cows than in adult cows (*p* < 0.05). Interestingly, fat and total solids were higher in 15 M dams (*p* < 0.05). On the contrary, protein concentration was highest in the 27 M group (*p* = 0.007).

**Figure 2:**
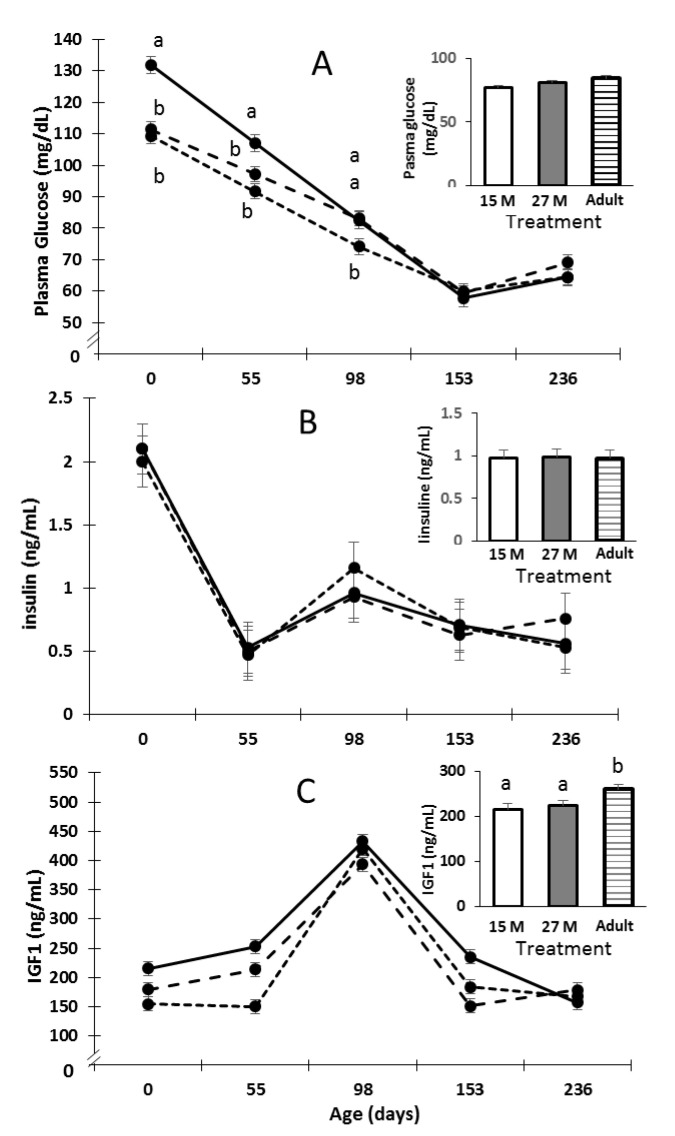
Glucose (**A**), serum insulin (**B**), and serum IGF1 (**C**) concentrations in calves from heifers impregnated at 15 months (••••), 27 months (– – –) and multiparous cows (–––), from birth until weaning. Different letters indicate that the difference between means at a specific time point is significant (*p* < 0.05) between treatment offspring. Values are means ± SEM. For glucose: treatment, *p* = 0.03; time, *p* < 0.0001; treatment × time, *p* = 0.05. For serum insulin: treatment, *p* = 0.99; time, *p* < 0.0001; treatment × time, *p* = 0.98. For serum IGF1: treatment, *p* = 0.01; time, *p* < 0.0001; treatment × time, *p* = 0.34.

Calves born from adult dams had higher fasting blood glucose concentration compared to calves from 15 M and 27 M dams from birth to 55 d and to 89 days of age, respectively (*p* < 0.05; Figure 2A). After 55 days of age, calves from 27 M and adult dams had higher glucose concentration with respect to 15 M dams, but no differences between groups were observed from 153 to 236 days of age. Serum insulin concentrations from birth until weaning at 236 d of age were not affected by treatment (*p* = 0.99; Figure 2B). Serum IGF1 concentration was increased in calves from adults cows compared to that in calves from 15 M and 27 M dams from birth to weaning (*p* = 0.01; Figure 2C).

## 4. Discussion

Age of dams at gestation has been suggested to influence the nutritional environment of the embryo and fetal growth in several species [5,6]. A retrospective cohort study of approximately four million nulliparous pregnant women <25 years of age showed that young maternal age is associated with increased risk of low birth weight babies independently of socioeconomic status, prenatal care and weight gain during pregnancy [19]. Studies in sheep have shown that juvenile ewes reduce nutrient delivery to their fetus and in fact shuttle nutrients towards their own body growth [20,21]. In addition, Kamal et al. [22] suggested that the intrauterine environment could limit fetal growth due to nutrient partition in growing ewes. In the bovine species, it has been well-documented that heifers and young cows generate smaller calves at birth and weaning than older cows do [7,23]. However, the effects of heifer age at first gestation on fetal and postnatal growth of progeny has been scarcely reported in beef cow fetal programming studies. In the present study, 15 M heifers reached 73% of adult BW at the beginning of the experiment, and had calves 2.3 and 5.0 kg lighter at parturition than calves from 27 M and adult dams, respectively. Dams that were bred at 27 months of age reached 90% of adult BW at the beginning of the experiment, and their calves were 2.7 kg lighter than calves from adult cows. The progeny from immature dams are generally 10 to 15% lighter at birth (e.g., in lambs, calves, piglets, and foals) compared with offspring born from dams of mature adult BW [7,24,25]. The partition of nutrients in the 15 M and 27 M heifers that were still growing could have affected the parturition of nutrients to the fetus depending on the age of the mother and caused the differences observed in the birth weight. The lack of BW gain in the 15 M heifers from day 107 to parturition could have influenced the results of this study.

Intrauterine growth retardation can be reflected in several morphometric changes in calves at birth [26]. In the current experiment, head circumference, body length and height were greatest in calves form adult dams. No differences were observed in body dimensions between calves from 15 M and 27 M mothers, except for heart girth where calves from 15 M cows were smaller than those from 27 M and adult cows. Nevertheless, fetal growth retardation has been associated with disproportionate organ growth [27,28]. Our study demonstrated that fetal growth can be affected disproportionately by the age of the mothers at gestation. Under conditions of fetal growth retardation, the development of the fetal brain is prioritized over other organs such as skeletal muscle, liver or kidney. This phenomenon is known as brain sparing [29]. Greater head circumference/BW ratio in newborn calves from 15 M and 27 M cows compared with those from adult cows suggests that brain sparing occurred. Sharma et al. [15] observed a similar effect in newborn lambs that developed in a restricted uterine environment. The reduced body mass index observed in calves from 15 M and 27 M mothers also suggests that they may have suffered growth retardation in utero.

Milk production was affected by dam age, and was in accordance with previous reports in multiparous beef cattle [14,30,31,32] and heifers [12]. In the present study, heifers produced 28.8% less milk than multiparous cows. Hansen et al. [33] evaluated the milk production in beef cows, and they concluded that cows in their third lactation produced about 30% more than the same cows during their first lactation. Milk production increased until beef cows reached 5 to 8 years of age, after which it decreased [34]. Clutter and Nielsen [35] evaluated the milk production for dams of various ages, and they reported that milk production was 25% higher in mature cows (4 to 5 years old) compared with primiparous cows. These reductions concur with the [36] model that uses a 26% reduction in milk yield for primiparous 2-year-old cows vs. mature cows. However, these differences may be greater depending on the quality of the diet, as Fox et al. [37] and Johnson et al. [38] reported. These authors used low-quality forage and found that primiparous cows produced 60% and 43% less milk, respectively, than multiparous cows. It is possible that the relatively low forage quality used in these experiments, and the resulting low energy intake, limited milk production of primiparous cows more than that of multiparous cows. According to NRC [36], adult cows are expected to have higher milk yield compared to heifers as a result of a higher net energy balance for milk production because heifers use dietary energy to fulfill the nutritional requirement for growth in addition to the maintenance and milk production requirements. There is a lack of milk production data for heifers of different ages at first calving. Nevertheless, in the present work, no differences in milk production between heifers of different ages at the first calving were found.

Many authors have reported that calf weaning weight is highly correlated with milk yield [15,39,40]. In this research, adult cows produced more milk than heifers (15 M and 27 M), and their calves were significantly heavier at weaning. The 15 M cows produced similar amounts of milk as 27 M cows, but the weight gains of the 15 M calves were significantly lower than those born from 27 M cows. Liu et al. [41], evaluating the relationship between preweaning calf growth and the dam´s milk production and composition, observed that some of the milk quality traits approached the same importance as milk quantity. Brown and Brown [42] found interactions between the preweaning ADG with milk fat and protein yield. Beal et al. [43] reported milk fat intake was positively associated with calf preweaning gain. Marston et al. [44] reported a greater association of absolute amounts of milk fat to adjusted weaning weight in Angus cows. However, in our study, 15 M cows had a higher fat percentage in their milk, but their calves had a lower weight gain, suggesting that total milk production is crucial to the resulting weaning weight of the calf. Results from Beal et al. [43] showed milk protein intake by calves to be positively associated with preweaning gain. In our study, the milk from 27 M cows had a higher percentage of protein than that from 15 M cows, and this could explain the greater weight gain of the calves from the 27 M cows compared to those from the 15 M cows. The lower pre-weaning weight gain of the calves from 15 M cows may also be a consequence of uterine growth retardation, which can negatively impact calves’ postnatal growth.

Prenatal and early postnatal nutrition can alter the ability of calves to regulate blood glucose concentration by altering pancreatic function or composition. Pancreas development is critical during late gestation and the first months after birth [45]. Experimentally induced intrauterine growth retardation in rats resulted in alterations of the endocrine pancreas, reduced pancreatic weight and β cell mass at birth, and lower insulin secretion in adult life. Similarly reduced fetal pancreatic β cell mass and increased β cell apoptosis have been observed in bovine fetuses that have been exposed to maternal nutrient restriction [46]. In our study, calves born from 15 M dams had reduced glucose during the first 98 days of life, and calves born from 27 M dams had reduced glucose during the first 55 days of life compared to calves from adult cows. The reduced glycemia observed in the first days of life in primiparous cows could reflect retarded pancreas development caused by a restrictive maternal nutrient environment during gestation or low milk consumption during lactation. Human studies have been focused on maternal age as a factor associated with low birth weight and increased prevalence of impaired glucose metabolism. Mothers under 24 years of age have a higher risk of low birth weight compared to mothers over 24 years of age [47]. Mericq et al. [48] observed that infants small for their gestational age showed increased insulin sensitivity at birth than did average-size infants. The low glucose concentration in calves from primiparous cows and similar insulin concentration in calves from adult cows could be related to higher insulin sensitivity during the first month of life. Gardner et al. reported that nutrient-restricted ewes during late gestation produced lambs with increased concentrations of insulin and plasma glucose after IVGTT [49]. Ford et al. reported that ewes restricted during early to mid-gestation with 50% of nutritional requirements, produced offspring with greater concentrations of plasma glucose in response to IVGTT at 63 and 250 days of age [50]. However, in bovine species, Maresca et al. reported that the rate of blood glucose clearance in steers at 24 months of age was correlated with protein availability of dams during late gestation [51]. Similar results were reported by Long et al. in female and male progeny at 15 months of age when dams were exposed to 55% of their nutritional requirement during early gestation [52]. The differences observed between species in the ability to remove blood glucose concentration after glucose infusion may be related to the physiological age of the offspring at the time of glucose tolerance testing. In this experiment, glucose concentrations after 98 days of age in calves from 27 M dams and after 153 days in calves from 15 M dams were similar to the concentrations in calves from adult cows. This could be indicative of differential capacity of pancreas compensatory development between primiparous cows of different ages.

The effects of a restrictive nutrient environment on fetal development may be mediated through alterations in the IGF axis [9]. The IGF1 and IGF2 are mitogenic peptides that have a fundamental role in the regulation of fetal growth due to their ability to stimulate proliferation and differentiation of multiple cell types [53]. Studies have shown that fetal serum IGF1 concentration is positively correlated to fetal weight, growth rate, crown–rump length and hip height in cattle [54], and birth weight in sheep [55]. Concentration of IGF1 positively modulates the protein synthesis rate and inhibits protein degradation rates contributing to myofiber hypertrophy during late gestation and postnatal growth [56]. It has been shown that nutrient restriction produces a decrease in serum IGF1 concentration, and that that is crucial to fetal growth [57]. Nutritional partition in primiparous young cows that are ending their own growth may reduce placenta size [58] and result in nutrient restriction to the fetus [59]. This nutrient restriction may result in the lower birth weights observed in the present results, as well as the decreased IGF1 concentrations, at birth and during lactation, in calves from 15 M and 27 M cows. In turn, decreased IGF1 in the offspring during lactation in association with lower milk production of the cows would result in lower rates of growth in these calves.

## 5. Conclusions

Numerous studies have been focused on determining the optimum reproductive development of beef heifers to ensure maximum pregnancy rates. However, offspring performance of first breed heifers has not been considered to define reproductive management strategies of replacement heifers. To the authors´ knowledge, this is the first study to determine the effects of dam age at first gestation on fetal and postnatal growth in beef cattle. It is clear that under conditions of extensive grazing in Argentinian cow-calf operations, first breed heifers produce lighter progeny at harvest than mature cows. Moreover, calves from 15 M heifers were lighter at weaning than calves from 27 M heifers. Differences observed in this study on postnatal growth performance, glucose metabolism and IGF1 concentration between 15 M and 27 M calves suggest that studies should be conducted in order to maximize not only the heifer pregnancy rate but also offspring performance. Considering the magnitude of calf harvests from first breed heifers in a typical cow-calf operation, more studies are needed to determine which mechanisms modify postnatal growth in the progeny and design nutritional strategies to minimize the differences in fetal and postnatal growth between the progeny of heifers and mature cows.

## Figures and Tables

**Table 1:** Effect of dam age on BW and BCS.

	Treatments ^1^				
Item	15 M	27 M	Adult	SEM	*p*-Value
BW, kg					
Initial ^2^	320 a	394 b	437 c	8.6	<0.001
at calving	343 a	423 b	504 c	7.7	<0.001
at weaning	321 a	383 b	450 c	8.5	<0.001
BCS ^3^					
Initial	5.3 a	5.6 a	4.8 b	0.07	<0.001
at calving	5.4 a	5.4 a	5.0 b	0.04	0.03
at weaning	4.0 a	4.2 a,b	4.4 b	0.06	0.02

^1^ 15 M = heifers that received AI at 15 months of age, 27 M = heifers that received AI at 27 months of age, Adult = multiparous cows. ^2^ Time point of 107 d before calving date. ^3^ Scale = 1 to 9 [13]. a,b,c Rows that do not have a common superscript differ, *p* < 0.05.

**Table 2:** Effect of dam age on calf performance from birth to weaning.

	Treatments ^1^				*p*-Value		
Item	15 M	27 M	Adult	SEM	Treat ^2^	Sex ^3^	Treat x Sex
Gestation length, d	282.8 a	282.1 a	279.6 b	0.75	0.009	0.30	0.38
BW at birth, kg	28.0 a	30.3 b	33.0 c	0.96	0.002	<0.001	0.60
BW at weaning, kg	158.1 a	181.1 b	206.4 c	5.2	<0.001	0.08	0.78
ADG, kg/d	0.590 a	0.680 b	0.790 c	0.02	<0.001	0.32	0.62
Head circ. ^4^, cm	45.7 a	45.9 a	47.2 b	0.44	0.03	0.15	0.27
Heart girth, cm	71.8 a	74.2 b	75.3 b	0.7	0.009	0.01	0.39
Cannon circ. ^4^, cm	11.7	11.5	12.0	0.2	0.15	0.004	0.84
Body length, cm	72.1 a	70.3 a	76.2 b	1.4	0.01	0.90	0.13
Height, cm	70.4 a	70.7 a	73.9 b	1.1	0.03	0.54	0.85

^1^ 15 M = heifers that received AI at 15 months of age, 27 M = heifers that received AI at 27 months of age, Adult = multiparous cows. ^2^ Treat = treatment. ^3^ 15 M = 11 females, 9 males, 27 M = 9 females, 11 males; Adult = 10 females, 10 males. ^4^ Circ. = circumference. a,b,c Rows that do not have a common superscript differ, *p* < 0.05.

**Table 3:** Effect of dam age on body measurements/birth weight ratio.

	Treatments ^1^				*p*-Value		
Item	15 M	27 M	Adult	SEM	Treat ^2^	Sex ^3^	Treat x Sex
Head cir. ^4^/birth BW, cm/kg	1.6 a	1.5 b	1.4 c	0.04	0.005	0.0008	0.92
Heart girth/birth BW, cm/kg	2.6 a	2.5 a	2.3 b	0.06	0.01	0.001	0.84
Cannon cir. ^4^ /birth BW, cm/kg	0.42 a	0.39 b	0.37 b	0.01	0.002	0.03	0.41
Body length/birth BW, cm/kg	2.6 a	2.3 b	2.4 b	0.07	0.04	0.0009	0.69
Height/birth BW, cm/kg	2.6 a	2.4 a	2.3 b	0.05	0.001	<0.001	0.41
Body mass index	3.3 a	3.5 a	3.8 b	0.09	0.002	0.0002	0.66

^1^ 15 M = heifers that received AI at 15 months of age, 27 M = heifers that received AI at 27 months of age, Adult = multiparous cows. ^2^ Treat = treatment. ^3^ 15 M = 11 females, 9 males, 27 M = 9 females, 11 males; Adult = 10 females, 10 males. ^4^ Cir. = circumferences. a,b,c Rows that do not have a common superscript differ, *p* < 0.05.

**Table 4:** Effect of dam age on milk composition.

	Treatments ^1^						
Item	15 M	27 M	Adult	SEM	Treat ^2^	Period	Treat × Period
Fat, g/100 mL	3.7 a	2.9 b	2.9 b	0.1	<0.0001	<0.0001	0.11
Protein, g/100 mL	3.28 a	3.45 b	3.34 a	0.06	0.007	<0.0001	0.11
Urea, mg/dL	12.5 a	12.7 a	11.6 b	0.3	0.04	<0.0001	0.36
Lactose, g/100 mL	4.4 a	4.3 a	4.6 b	0.06	0.03	<0.0001	0.30
Total solids, g/100 mL	12.4 a	11.8 b	11.8 b	0.2	<0.0001	0.0002	0.45

^1^ 15 M = heifers that received AI at 15 months of age, 27 M = heifers that received AI at 27 months of age, Adult = multiparous cows. ^2^ Treat= treatment. a,b,c Rows that do not have a common superscript differ, *p* < 0.05.

## Data Availability

The data are available by contacting the corresponding author.
